# A Novel Two-Axis Load Sensor Designed for *in Situ* Scratch Testing inside Scanning Electron Microscopes

**DOI:** 10.3390/s130202552

**Published:** 2013-02-18

**Authors:** Hu Huang, Hongwei Zhao, Boda Wu, Shunguang Wan, Chengli Shi

**Affiliations:** College of Mechanical Science & Engineering, Jilin University, Changchun 130025, China; E-Mails: huanghuzy@163.com (H.H.); wubd@mail.cdgdc.edu.cn (B.W.); wanshunguang@126.com (S.W.); shichengli163@163.com (C.S.)

**Keywords:** load sensor, *in situ* scratch testing, decoupling algorithm, frequency response, calibration experiment

## Abstract

Because of a lack of available miniaturized multiaxial load sensors to measure the normal load and the lateral load simultaneously, quantitative *in situ* scratch devices inside scanning electron microscopes and the transmission electron microscopes have barely been developed up to now. A novel two-axis load sensor was designed in this paper. With an I-shaped structure, the sensor has the function of measuring the lateral load and the normal load simultaneously, and at the same time it has compact dimensions. Finite element simulations were carried out to evaluate stiffness and modal characteristics. A decoupling algorithm was proposed to resolve the cross-coupling between the two-axis loads. Natural frequency of the sensor was tested. Linearity and decoupling parameters were obtained from the calibration experiments, which indicate that the sensor has good linearity and the cross-coupling between the two axes is not strong. Via the decoupling algorithm and the corresponding decoupling parameters, simultaneous measurement of the lateral load and the normal load can be realized via the developed two-axis load sensor. Preliminary applications of the load sensor for scratch testing indicate that the load sensor can work well during the scratch testing. Taking advantage of the compact structure, it has the potential ability for applications in quantitative *in situ* scratch testing inside SEMs.

## 1. Introduction

In recent years, indentation testing and scratch testing have been very important methods to characterize the mechanical properties of materials, especially for micro/nano materials and structures, thin films and coatings, and they have been widely used in the fields of material science, semiconductors, nanotechnology, biomechanics and so on [1–5]. By applying normal loads on the surface of materials, indentation testing is mainly used to evaluate mechanical properties of materials such as hardness and elastic modulus [6]. In contrast with indentation testing, scratch testing is mainly used to study the abrasion resistance of bulk materials and adhesion strength of thin films by the process whereby an indenter scratches the sample surface [7–10], and it has a more complex contact process. Compared with conventional *ex situ* indentation and scratch testing, *in situ* indentation and scratch testing inside the scanning electron microscope (SEM) and the transmission electron microscope (TEM) have the function of dynamically observing the contact process between the indenter and the sample [11–18], which is meaningful to investigate deformation and damage mechanisms of materials during the indentation and scratch testing process and to explain discontinuous phenomena appearing in the penetration load-depth curves. The design of *in situ* indentation and scratch devices compatible with the SEM and TEM has limitations arising from the characteristics of the SEM and TEM, such as the small volume of the chamber, short working distance, electromagnetic sensing, the vacuum environment and vibration sensing [16]. Up to now, quantitative *in situ* SEM and TEM indentation devices have been presented by researchers and some of them can also carry out *in situ* scratch testing inside the SEM qualitatively [14,18]. However, the quantitative *in situ* scratch device inside the SEM and TEM is seldom discussed because of a lack of available miniaturized multiaxial load sensors to measure the normal load and the lateral load synchronously.

Based on the principle of strain measurement, various kinds of load sensors, uniaxial sensors and multiaxial sensors were developed by previous researchers for different applications [19–23]. Some applications of strain gauges inside the SEM [14,24] indicate that it is feasible to realize precision measurement inside the SEM via strain gauges. In this paper, a novel two-axis load sensor with a compact structure was designed based on the principle of strain measurement. The structure and the measurement principle were introduced. A decoupling algorithm was proposed to resolve the cross-coupling between the two axes. Stiffness and modal characteristics of the elastic body were analyzed by the finite element method. Experiments were carried out to evaluate the performances of the sensor. Preliminary applications of the load sensor for scratch testing indicate that the load sensor can work well during the scratch testing. The sensor has the potential application for *in situ* scratch testing inside the SEM because of the compact structure.

## 2. Structure and Principle of the Sensor

**Figure 1.**
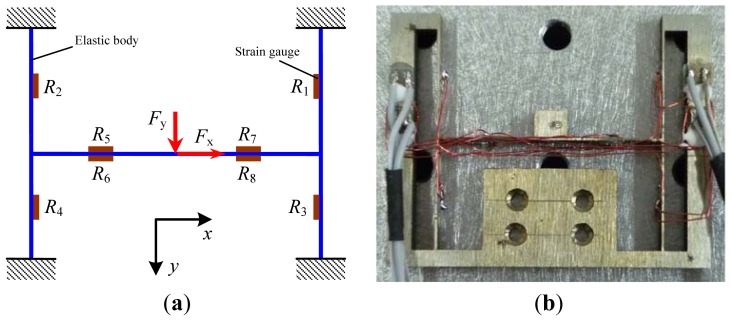
The schematic diagram (**a**) and the corresponding prototype (**b**) of the sensor.

Considering the small volume and short working distance of the SEM, design of the elastic body is the key for the load sensor, requiring the function of two-axis measurement and a miniaturized structure. A schematic diagram and the corresponding prototype of the sensor are shown in Figure 1(a,b), respectively. The load sensor with the dimensions of 58 mm × 46 mm × 5 mm mainly consists of the elastic body and eight strain gauges. The elastic body with the I-shaped structure was processed by wire cutting using the material 65 Mn. The strain gauges are BFC-350-3AA-11 type and the grid material is constantan with a resistance of 350 Ohm. The strain gauges are adhered onto the surface of the elastic body with M-Bond 610 adhesive. These eight gauges can be divided into two groups. The first group on the two sides of the elastic body consisting of strain gauges with resistances of *R*_1_, *R*_2_, *R*_3_ and *R*_4_ is mainly used to measure the lateral load during the scratch testing. The second group on the middle of the elastic body consisting of strain gauges with resistances of *R*_5_, *R*_6_, *R*_7_ and *R*_8_ is mainly used to measure the normal load during the scratch testing.

**Figure 2.**
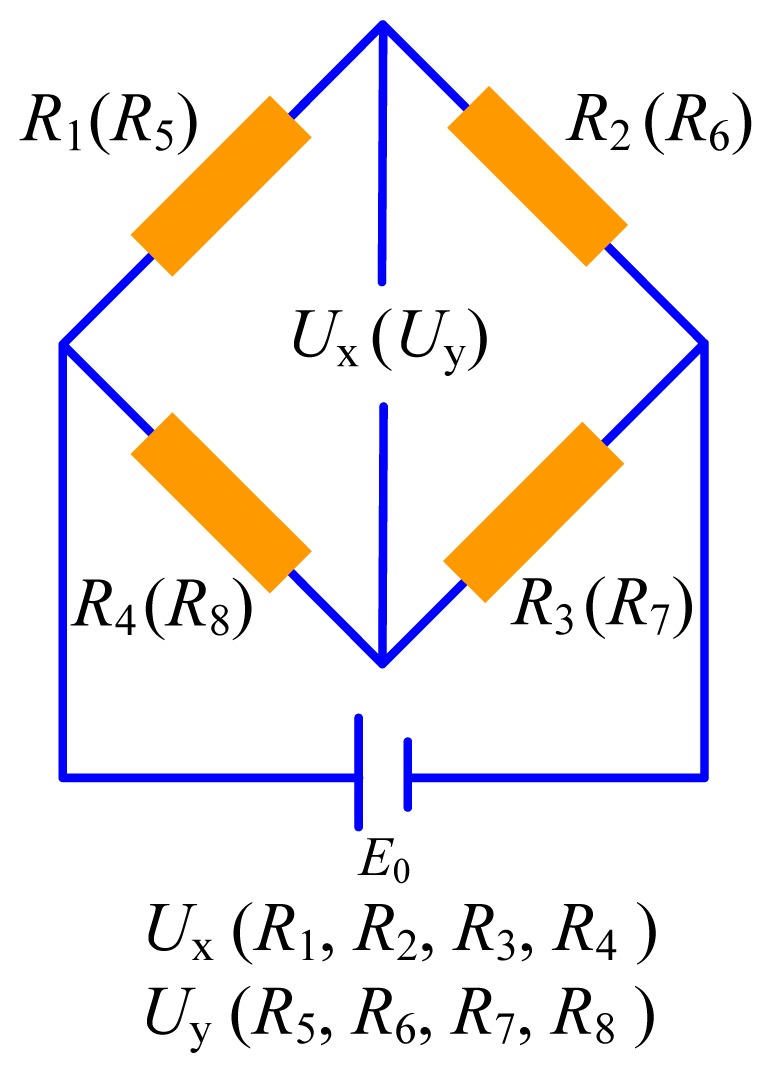
The Wheatstone bridge.

Figure 2 is the schematic diagram of the Wheatstone bridge that converts the resistance change of the strain gauges to voltage change.

**Figure 3.**
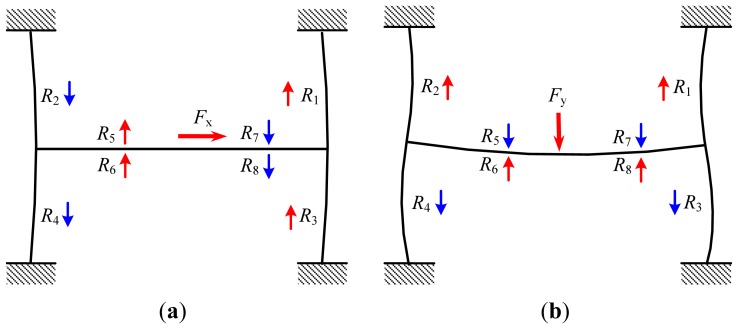
Deformation diagrams of the elastic body under (**a**) lateral load *F*_x_ and (**b**) normal load *F*_y_.

In order to describe the principle of the load sensor better, the deformation diagrams of the elastic body under the lateral load and the normal load are illustrated as shown in Figure 3. As seen in Figure 3(a), when the lateral load is applied on the sensor, two sides of the elastic body will bend and the middle of the elastic body will be tensile and compressed, which leads to a resistance increase of the strain gauges with the initial resistances of *R*_1_, *R*_3_, *R*_5_ and *R*_6_ but a resistance decrease of the strain gauges with the initial resistances of *R*_2_, *R*_4_, *R*_7_ and *R*_8_. According to Figure 2, the voltage output of the first group is obvious, but the voltage output of the second group is nearly zero, which indicates that strain gauges on the two sides of the elastic body are sensitive to the lateral load but strain gauges on the middle of the elastic body are less sensitive to the lateral load. So, strain gauges on the two sides of the elastic body can be used to measure the lateral load.

As shown in Figure 3(b), when a normal load is applied to the sensor, resistances of the strain gauges with the initial resistances of *R*_1_, *R*_2_, *R*_6_ and *R*_8_ will increase but resistance of the strain gauges with the initial resistances of *R*_3_, *R*_4_, *R*_5_ and *R*_7_ will decrease. According to Figure 2, the voltage output of the second group is obvious but the voltage output of the first group is nearly zero, which indicates that strain gauges on the middle of the elastic body are sensitive to the normal load but strain gauges on the two sides of the elastic body are less sensitive to the normal load. So, strain gauges on the middle of the elastic body can be used to measure the normal load.

## 3. Finite Element Analysis

Stiffness of the elastic body affects the dynamic response of the sensor and total compliance of the scratch device. Stiffness and modal characteristics of the elastic body were analyzed by the finite element method. In order to make the simulation closer to the true situation, the indenter was added on the middle of the sensor during the analysis.

**Figure 4.**
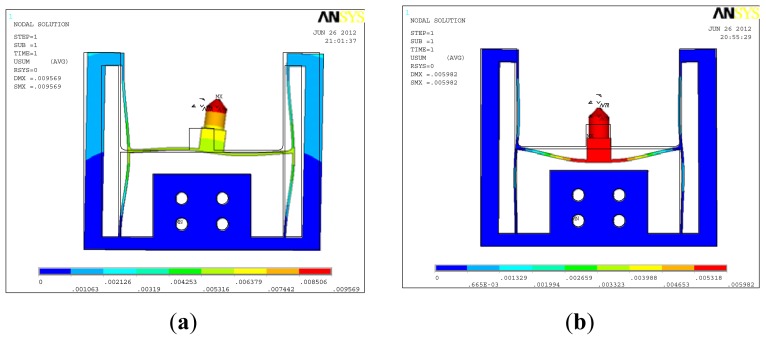
Deformation of the elastic body including the indenter (**a**) under the lateral load of 1 N and (**b**) under the normal load of 1 N.

For stiffness analysis along the *x* axis, a load of 1 N was applied on the tip of the indenter and the deformation result is shown in Figure 4(a). The deformation of the elastic body is similar to Figure 3(a), but a difference is also observed because of existence of the equivalent moment coming from the indenter height. The tip of the indenter has the maximum deformation of 9.57 μm, and the stiffness of the elastic body along the *x* axis is 9.57 nm/mN. Similarly, the tip of the indenter has the maximum deformation of 5.98 μm under the load of 1 N applied on the middle of the elastic body along the *y* axis, and the stiffness of the elastic body along the *y* axis is 5.98 nm/mN.

**Figure 5.**
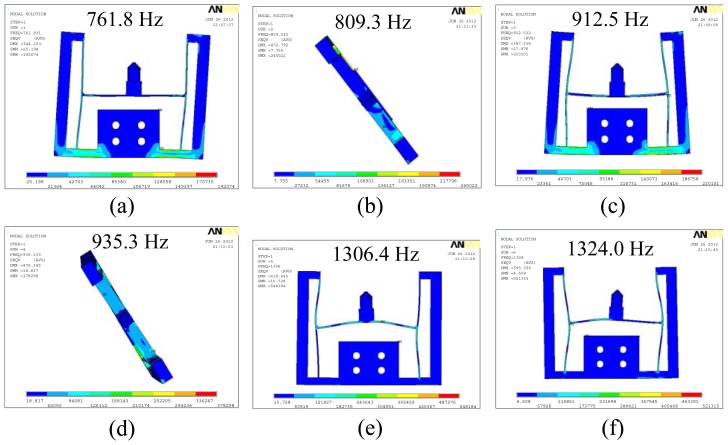
The first six order mode shapes of the elastic body including the indenter.

Modal analysis is an effective method to evaluate dynamic performances of mechanical structures and systems. Modal analysis of the elastic body including the indenter was carried out and the first six order mode shapes of the structure are given in Figure 5, corresponding to the first six order natural frequencies of 761.8, 809.3, 912.5, 935.3, 1,306.4 and 1,324.0 Hz, respectively. Because of the high first order frequency of 761.8 Hz, the sensor has potential applications under high frequency conditions.

## 4. Decoupling Algorithm

As mentioned in Section 2, strain gauges on the two sides of the elastic body and strain gauges on the middle of the elastic body are mainly used to measure the lateral load and the normal load, respectively, but according to Figures 3 and 4, cross-coupling between the two axes also exists, which means that the lateral load also leads to the voltage output of the strain gauges on the middle of the elastic body especially when the indenter exists, and the normal load also leads to the voltage output of the strain gauges on the two sides of the elastic body, so how to distinguish the lateral load and the normal load from the measured output voltages is the key for using this two-axis load sensor.

Let *u*_xx_ and *u*_yx_ be the output voltages of the strain gauges on the two sides of the elastic body under per unit lateral load and per unit normal load, respectively. Let *u*_xy_ and *u*_yy_ be the output voltages of the strain gauges on the middle of the elastic body under per unit lateral load and per unit normal load, respectively. So, the measured voltages and the applied loads have the following relationships:

(1) $$U_{\text{x}}=F_{\text{x}}u_{\text{xx}}+F_{\text{y}}u_{\text{yx}}$$

(2) $$U_{\text{y}}=F_{\text{x}}u_{\text{xy}}+F_{\text{y}}u_{\text{yy}}$$

where *U*_x_ and *U*_y_ are the measured output voltages from the strain gauges on the two sides of the elastic body and the strain gauges on the middle of the elastic body respectively, and *F*_x_ and *F*_y_ are the applied lateral load and the normal load respectively.

According to Equations (1) and (2), the lateral load and the normal load can be given as:

(3) $$F_{\text{y}}=\frac{u_{\text{xx}}U_{\text{y}}-u_{\text{xy}}U_{\text{x}}}{u_{\text{xx}}u_{\text{yy}}-u_{\text{xy}}u_{\text{yx}}}$$

(4) $$F_{\text{x}}=\frac{u_{\text{yy}}U_{\text{x}}-u_{\text{yx}}U_{\text{y}}}{u_{\text{xx}}u_{\text{yy}}-u_{\text{xy}}u_{\text{yx}}}$$

In Equations (3) and (4), *U*_x_ and *U*_y_ can be measured directly, so the next step is to obtain the values of *u*_xx_, *u*_yx_, *u*_xy_ and *u*_yy_, named decoupling parameters, which can be resolved by calibrating the load sensor.

## 5. Experiments

### 5.1. Frequency Response

**Figure 6.**
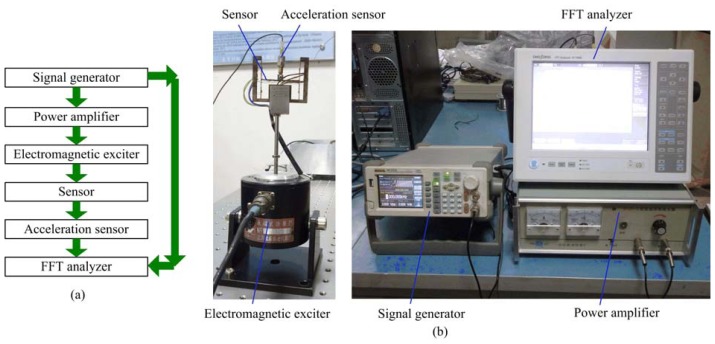
The experimental system.

Frequency response is an important characteristic to evaluate performances and applications of sensors. Frequency response of the two-axis load sensor was obtained from a dynamic signal analyzer through swept-sine analysis with frequencies spanning a range of 1,000 Hz. Figure 6 is the experimental system. Figure 6(a) is the experimental principle and Figure 6(b) gives detail of the experimental system. Swept-sine signal was generated by the signal generator and amplified by the power amplifier, and then sent to the electromagnetic exciter. Exciting force drove the two-axis load sensor to vibrate. Vibration acceleration was measured by the acceleration sensor (ONO SOKKI CO., LTD., NP-3414). Signals from the acceleration sensor and the signal generator were collected and analyzed by the Fast Fourier Transform (FFT) analyzer (ONO SOKKI CO., LTD., CF-7200A) synchronously.

**Figure 7.**
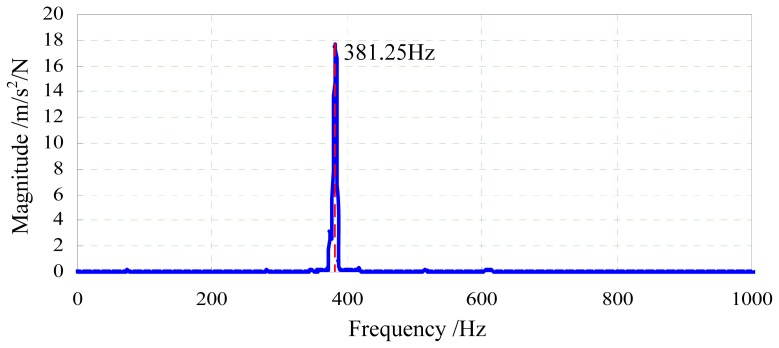
Frequency response of the two-axis load sensor.

Figure 7 is the experimental result. It can be seen that the first order natural frequency of the two-axis load sensor is 381.25 Hz, which is less than the calculation value of 761.8 Hz in the Figure 5. Reasons may be as follows: (1) the manufacturing error leads to dimensional errors between the analysis model and the prototype; (2) parameters used for simulation may be different from the actual materials; (3) the additional mass coming from the acceleration sensor and electrical wires is relatively large compared with the mass of the two-axis load sensor. Masses of the two-axis load sensor (including electrical wires), the indenter and the acceleration sensor are about 51.581 g, 0.821 g and 4.449 g, respectively, so the actual first order natural frequency of the two-axis load sensor is over 381.25 Hz.

### 5.2. Calibration of the Sensor

**Figure 8.**
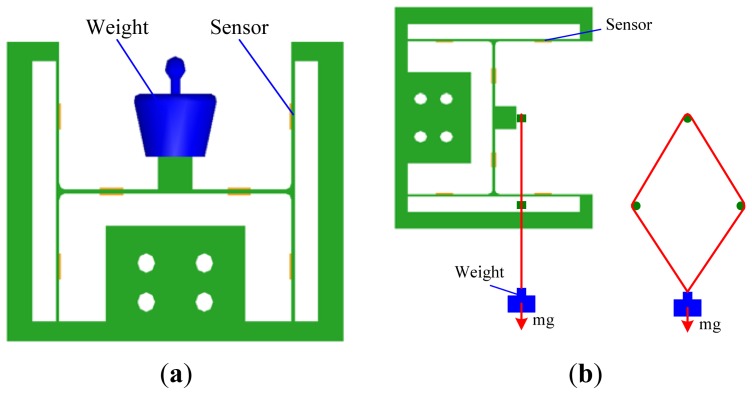
Loading programs for (**a**) the normal load and (**b**) the lateral load.

**Figure 9.**
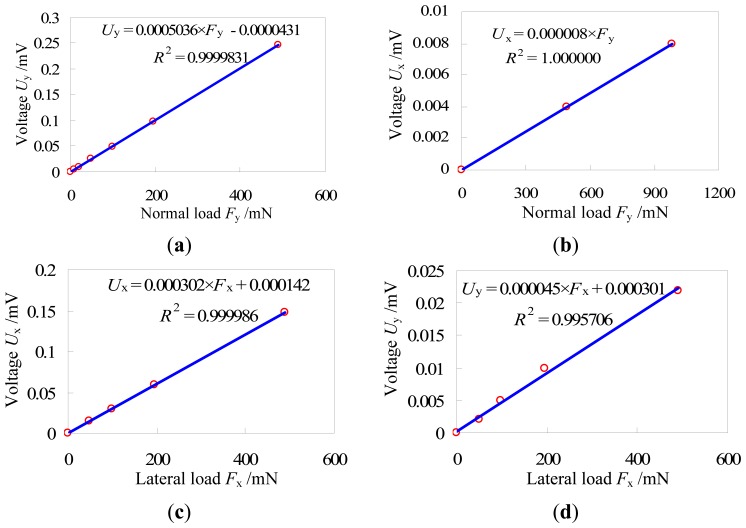
Calibration curves of the two-axis load sensor. (**a**) is the relationship between the normal load *F*_y_ and the output voltage *U*_y_ from the strain gauges on the middle of the elastic body; (**b**) is the relationship between the normal load *F*_y_ and the output voltage *U*_x_ from the strain gauges on the two sides of the elastic body; (**c**) is the relationship between the lateral load *F*_x_ and the output voltage *U*_x_ from the strain gauges on the two sides of the elastic body; (**d**) is the relationship between the lateral load *F*_x_ and the output voltage *U*_y_ from the strain gauges on the middle of the elastic body.

Calibration experiments of the two-axis load sensor were carried out to evaluate linearity of the sensor and to obtain the decoupling parameters. The loading programs are illustrated in Figure 8. As shown in this Figure, the normal load is easy to load by the standard weights but the lateral load is difficult to load because of potential interference when the lateral load is directly loaded like the normal load, so a parallelogram transformation mechanism as shown in Figure 8(b) was developed to load the lateral load. The output voltages were measured by a digital multimeter. The experimental results are shown in Figure 9. The least squares fitting method was used to obtain the relationship between the measured voltages and the applied loads. The linear correlation coefficients *R*^2^ are all close to 1, indicating that the load sensor has good linearity for two axes. Slopes of these fitted curves are the decoupling parameters, and values of *u*_xx_, *u*_yx_, *u*_xy_ and *u*_yy_ are 0.000302, 0.000008, 0.000045 and 0.005036 mV/mN, respectively. The difference between *u*_xx_ and *u*_yx_ is very large and also the difference between *u*_yy_ and *u*_xy_ is large, which indicate that the cross-coupling between the two axes is not strong and the I-shaped structure is reasonable and feasible.

According to the obtained decoupling parameters, Equations (3) and (4) can be rewritten as:

(5) $$F_{\text{y}}=198.62U_{\text{y}}-29.59U_{\text{x}}$$

(6) $$F_{\text{x}}=3312.04U_{\text{x}}-5.26U_{\text{y}}$$

Consequently, the normal load and the lateral load can be obtained simultaneously by Equations (5) and (6). Taking advantage of the compact structure, especially the small height, the developed two-axis load sensor has potential applications for *in situ* scratch testing inside the SEM.

## 6. Preliminary Applications for Scratch Testing

**Figure 10.**
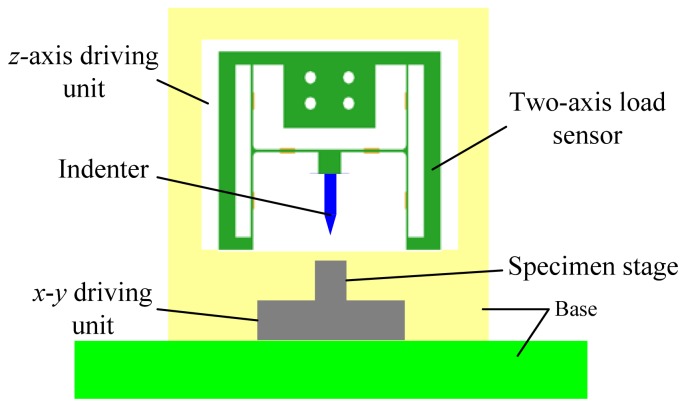
Schematic diagram for the application of conventional scratch testing.

**Figure 11.**
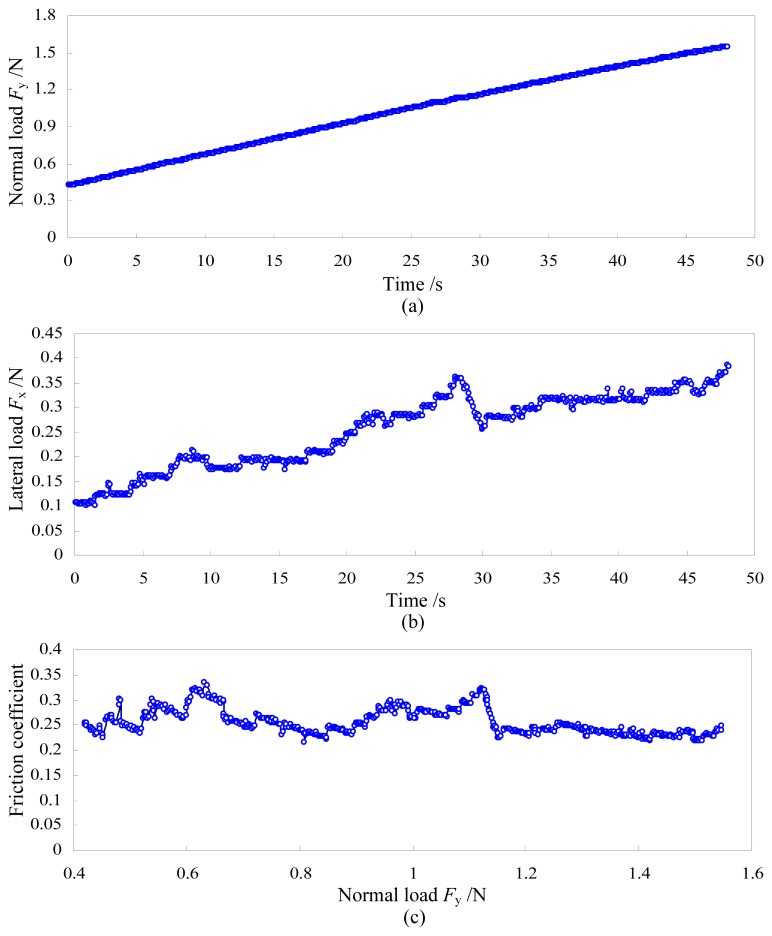
Scratch testing results of the Zr-based bulk metallic glass. (**a**) is the relationship between the normal load and time; (**b**) is the relationship between the lateral load and time; (**c**) is the relationship between the friction coefficient and the normal load.

In order to verify feasibility of the two-axis load sensor for applications of scratch testing, the experimental system for conventional scratch testing was established as shown in Figure 10. The *z*-axis driving unit is used to realize the penetration process of the indenter. The *x*-*y* driving unit is used to realize the scratch process, during which the normal load and the lateral load are measured by the two-axis load sensor. Scratch testing of the Zr-based bulk metallic glass was carried out using the diamond Vickers indenter after the load sensor was calibrated and decoupled. The scratch velocity is 30 μm/s and the scratch depth is not measured in current experiments. Experimental results are illustrated in Figure 11. In Figure 11(a), the normal load almost increases linearly with increasing of the scratch length while the lateral load fluctuates up and down as shown in Figure 11(b). The friction coefficient between the diamond Vickers indenter and the bulk metallic glass is obtained via dividing the lateral load by the normal load, and the relationship between the friction coefficient and the normal load is illustrated in Figure 11(c). In an approximation, the friction coefficient fluctuates up and down when the normal load is less than 1.15 N and it becomes relatively stable when the normal load is larger than 1.15 N.

**Figure 12.**
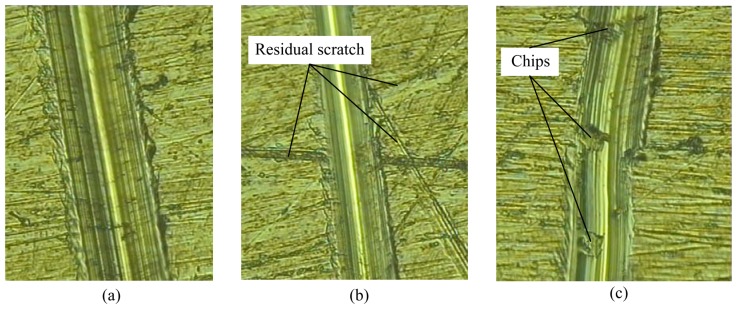
Residual scratch morphologies of the Zr-based bulk metallic glass.

In order to find the possible reasons for fluctuating of the lateral load and the friction coefficient during the scratch testing, the residual morphologies were measured by the optical microscope and the results are given in Figure 12. Figure 12(a) shows the relatively perfect residual scratch morphology when the indenter scratches on the surface with relatively good quality. Figure 12(b) shows the residual scratch morphology when the indenter passes the surface with residual scratch generated during the sample preparation. Figure 12(c) shows residual chips on the residual scratch morphology. The residual scratch generated during the sample preparation and formation, accumulation and fracture of chips during the scratch process are all potential reasons leading to fluctuation. Larger effects of these factors on the measured curves will appear when the normal load is smaller. Of course, fluctuation was also observed by previous literatures [25,26] but the real reasons have not been revealed up to now. The conventional scratch testing can obtain quantitative curves but cannot monitor the scratch process, and the current *in situ* scratch device can dynamically observe the scratch process but cannot measure the lateral load because there is no suitable load sensor.

**Figure 13.**
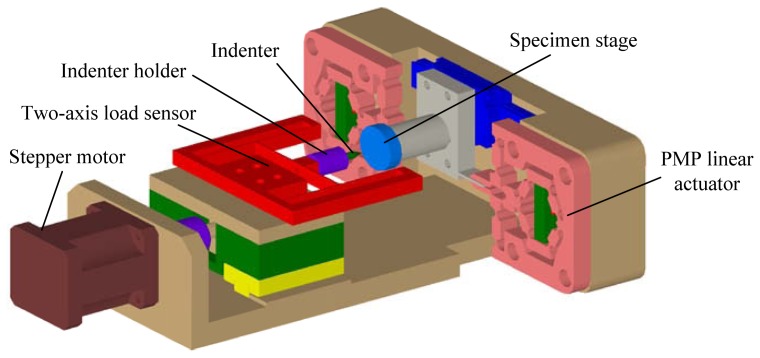
Preliminary model for the application of *in situ* scratch testing.

From Figures 11 and 12, we can conclude that the developed two-axis load sensor can work well during the scratch process and it can be used to realize conventional scratch testing. Taking advantage of the compact structure, it has potential applicability in quantitative *in situ* scratch testing inside the SEM, which is our future work. Here, the preliminary model for application of *in situ* scratch testing is given as shown in Figure 13. A stepper motor is used to realize penetration and withdrawing of the indenter. The parasitic motion principle (PMP) linear actuator [27] is used to realize the scratch process. During the scratch testing, the normal load and the lateral load are measured by the developed two-axis load sensor. As mentioned in Section 1, feasibility that using strain gauges to realize precision measurement inside the SEM has been verified in references [14,24]. However, some issues such as electromagnetic shield, thermal drift, cross talk and so on, should be solved before applications of the two-axis load sensor for *in situ* scratch testing inside the SEM. Issues mentioned above and design and experimental research on the designed *in situ* scratch device as shown in Figure 13 will be studied further and presented in another new paper.

## 7. Conclusions

In this paper, a novel two-axis load sensor was designed for future application in the field of *in situ* scratch testing inside a scanning electron microscope. The sensor based on the principle of strain measurement has an I-shaped structure, allowing it to measure the lateral load and the normal load simultaneously but still have a compact structure. The finite element method was used to analyze stiffness and modal characteristics of the elastic body including the indenter, and the simulation results indicate that stiffnesses of the elastic body along the *x* axis and the *y* axis are 9.57 nm/mN and 5.98 nm/mN, respectively, and the first order frequency of the sensor is about 761.8 Hz. A decoupling algorithm was proposed to resolve the cross-coupling between the two axes. The experimental system was established to test the frequency response of the sensor. Main error sources between the simulation result and the experimental result were analyzed, and the natural frequency of the sensor is over 381.25 Hz. Calibration experiments were carried out to evaluate the performance of the sensor. Results of these calibration experiments indicate that the load sensor has good linearity. Decoupling parameters are also obtained from the experiments. Analyzing the decoupling parameters, conclusion can be given that the cross-coupling between the two axes is not strong and the I-shaped structure is reasonable and feasible. According to the decoupling algorithm and the corresponding decoupling parameters, simultaneous measurement of the lateral load and the normal load can be realized.

The developed two-axis load sensor was used to carry out preliminary scratch testing of Zr-based bulk metallic glass. Experimental results indicate that the sensor can work well during the conventional scratch testing. Also, the preliminary model for application of *in situ* scratch testing was given. Of course, the developed two-axis load sensor cannot be directly used inside the scanning electron microscope because of existence of the strong electromagnetic field. Electromagnetic shielding will be necessary. Electromagnetic compatibility and cross talk of the sensor will be studied carefully in the future.
